# Recovery of Rose Flower Waste to Formulate Eco-Friendly Biopolymer Packaging Films

**DOI:** 10.3390/molecules28073165

**Published:** 2023-04-02

**Authors:** Nadka Tz. Dintcheva, Elisabetta Morici

**Affiliations:** 1 Dipartimento di Ingegneria, Università di Palermo, Viale delle Scienze Ed. 6, 90128 Palermo, Italy; 2 Advanced Technologies Network (ATeN) Center, Università di Palermo, Viale delle Scienze Ed. 18, 90128 Palermo, Italy

**Keywords:** biopolymer films, rose flower waste, materials circularity

## Abstract

Considering the circular principles of materials and investigating the possibility to use waste materials before their final disposal, in this work, dry rose flower (DRF) and rose flower waste (RFW), after oil extraction, have been considered as suitable materials for the formulation of biopolymer packaging films. Both DRF and RFW particles have been characterized by spectroscopy analysis, and their radical scavenger ability has been investigated. Moreover, DRF and RFW particles have been added by melt mixing to PolyLactic Acid (PLA), and formulated PLA-based films have been studied through rheology analysis, mechanical test, differential scanning calorimetry, and microscopy observations. Finally, the influence of both DRF and RFW particles on the photo-oxidation behavior of PLA has been evaluated by subjecting thin films to UVB exposure, and the progress of degradation has been monitored following the accumulations of oxygen-containing groups in time. Obtained results suggest that both DRF and RFW have a beneficial effect on the photo-oxidation behavior of PLA, and they can slow down PLA degradation upon UVB exposure. Therefore, PLA-based composite materials could be considered a good candidate for applications as packaging films.

## 1. Introduction

The European Union Green Deal focuses on a competitive bioeconomy for a sustainable future; there are two main principles: (i) the formulation and production of bio-inspired materials using natural feedstocks and (ii) the transition from linear to the circular economy, with the latter based increasingly on renewable energy and materials, accelerated by digital innovation. Therefore, the circular economy is a framework of three principles: (i) eliminate waste and pollution, (ii) keep products and materials in use, and (iii) regenerate natural systems [1,2,3].

In the last two decades, numerous scientific papers have reported the possibility to formulate composites using naturally occurring fibers and fillers and/or by-products coming from the agricultural and wood industry. As is widely documented, the presence of natural fibers and fillers in polymers and biopolymers could significantly increase some properties and performance, but unfortunately, in some cases, the naturally occurring fibers and fillers make the composites less resistant to oxidation phenomena and/or to degradation processes. In particular, according to the circular principles, different scientific papers investigated the possibility to formulate materials using agricultural waste and keep products and materials in use long before their final disposal and/or incineration [4,5,6,7,8,9,10,11,12,13,14]. However, as currently documented, the formulation of new-generation food packaging, based on biowaste materials and having antimicrobial and antioxidant properties, could be considered an innovative issue to produce low-cost active and sustainable packaging [13,14].

Since ancient times, the Bulgarian Rosa Damascena Mill has been used for essential oil extraction for cosmesis, perfumery, skincare, aromatherapy, pharmaceuticals, etc. As is well known, rose essential oils show a strong antioxidant capacity and antibacterial activity, and for this reason, over the millennia, roses have been subjected to extraction through distillation technologies at high temperatures [15]. Interestingly, to improve the essential oils’ yield and quality, the extraction process has been currently performed by also considering innovative subcritical or supercritical fluids [16,17]. After essential oil extraction, the dried rose flower waste has been successfully considered a food additive in animal husbandry (e.g., pigs, broilers, and lambs) or phytonutrients. However, the accurate compositional analysis of dried rose flower waste has been carried out through spectrometry and spectroscopy techniques to evaluate the suitability as a food additive for animal husbandry, increasing their growth performance [15,16,17,18,19,20].

As documented by Schieber et al., the flavonol glycosides extracted from distilled rose petals of oil-bearing rose contain twenty-two major components, including kaempferol and quercetin glycosides, quercetin 3-O-galactoside, and quercetin 3-O-xyloside. Therefore, accurate LC-MS analysis identified 13 kaempferol derivates in ca. 80% *v/v* and 11 quercetin glucosides in 20% *v/v*, which were water-ethanolic extract from industrial distilled rose petals [20].

A currently published paper by Dragoev et al. concluded that dry distilled rose petals contain three groups of polyphenol compounds: glycosides of kaempferol > glycosides of quercetin > glycosides of gallic acid, which showed high antioxidant activity. Moreover, the authors asserted that dried rose petals could be considered suitable food additives for pig husbandry. [19]

Considering circular economy principles, the possibility to use dry rose flower and rose flower waste, specifically, Bulgarian Rosa (Rosa Damascena Mill.), for the formulation of eco-friendly biopolymer packaging films, which could be considered a suitable method for reusing rose waste, before their disposal, is investigated. Rose flower waste (RFW), after essential oil extraction, was subjected to milling, and obtained particles were introduced in polylactic acid (PLA) by melt mixing to formulate packaging films. The antioxidant activity of dry rose flower and rose flower waste particles was characterized by a DPPH test, and the obtained results suggested good ability in free radical scavenging of the particles. Formulated PLA-based films show good mechanical performance and increased photo-oxidation resistance due to the presence of both dry rose flowers and rose flower waste.

## 2. Results

### 2.1. Characterization of Dry Rose Flower (DRF) and Rose Flower Waste (RFW)

**Figure 1:**
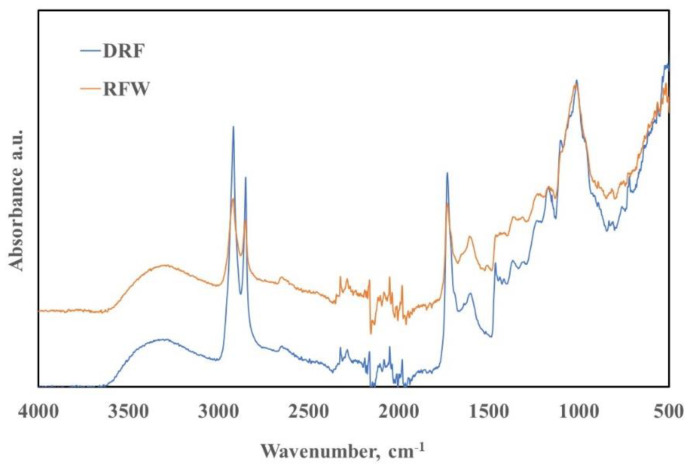
ATR-FTIR spectra of dry rose flower (DRF) and rose flower waste (RFW), before and after essential oil extraction, respectively.

Both dry rose flower (DRF) and rose flower waste (RFW) have been characterized by ATR-FTIR analysis, and in Figure 1, the ATR-FTIR spectra of both DRF and RFW are plotted. It is worth noting that the spectra of DRF and RFW are very similar, although some small differences can be observed regarding the peaks at 2917, 2849, and 1728 cm^−1^. Particularly, the intensities of these peaks are more pronounced for DRF rather than for RFW. As is widely known, these peaks could be attributed to the asymmetric and symmetric vibration of -CH groups (2917 and 2849 cm^−1^), and oxygen-containing groups (1728 cm^−1^). According to the literature, essential-oil roses contain numerous different constituents (more than 40), and, obviously, the ATR peaks appear very complex and the assignment of them is not exactly easy. Beyond this, the small observed differences in peak intensities of DRF and RFW could be understood considering that some essential oils and antioxidant moieties are presented to a limited extend in RFW, due to essential oil extraction. Therefore, the cellulose and lignin-based constituents could have slightly changed during oil extraction at high temperatures, but they are presented in both DRF and RFW.

Furthermore, in Table 1, the results of CHN test and particles dimension, after milling, are reported. As expected, the carbon contents of RFW is slightly higher that of DRF, probably, because some oils constituents have been subtracted during essential oil extraction. The hydrogen and nitrogen amounts are slightly higher in DRF rather than in RFW.

The particle dimensions, after milling, of both DRF and RFW are very similar, and this could be considered a good assumption in using RFW as suitable particles for the formulation of sustainable biopolymer composites.

**Figure 2:**
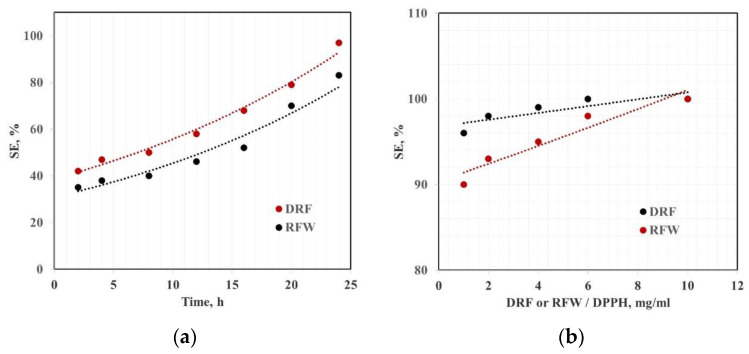
Scavenging efficiency of dry rose flower (DRF) and rose flower waste (RFW) of (**a**) 1 mg/mL of solid as a function of time (left) and (**b**) varying the content of solid in DPPH solution after 24 h (right).

The radical scavenging efficiency of both DRF and RFW is evaluated by DPPH (1,1-diphenyl-2-pycryl) test, and the obtained results are plotted in Figure 2a,b. Specifically, DRF and RFW have been dispersed in methanol solution in the presence of DPPH radicals, and the chemical activity of both DRF and RFW has been evaluated by monitoring the drop in absorbance at 517 nm of each sample. In Figure 2a, the kinetic of scavenging activity as a function of time, keeping the same amount of DRF and RFW, has been evaluated, and it is noticeable that both DRF and RFW show very similar scavenging ability, although RFW is slightly kinetic in terms of scavenging activity. The maximum scavenging activity, after 24 h, for DRF could be achieved considering ca. 6 mg of DRF in 2 mL of DPPH solution, while RFW required ca. 10 mg to obtain maximum scavenging activity, see Figure 2b.

### 2.2. Characterization of PLA-Based Composites Containing DRF and RFW

**Figure 3:**
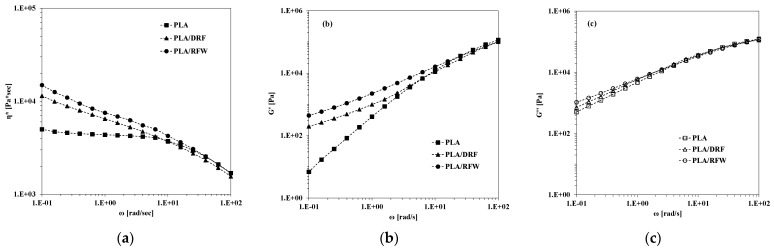
(**a**) Complex viscosity, η; (**b**) storage modulus, G’; and (**c**) loss modulus, G’’, of neat PLA and PLA containing 10%wt. of DRF and RFW.

The rheological behavior of neat PLA and PLA containing DRF and RFW has been evaluated, and trends of obtained complex viscosity, storage, and loss moduli, as a function of frequency, are plotted in Figure 3a–c. As can be noticed, neat PLA shows Newtonian behavior in a low-frequency range and well pronounced shear tinning in a high-frequency range, see Figure 3a. Due to the adding of both DRF and RFW, the viscosity increases significantly at low frequencies, and this effect if not appreciable at high frequencies. Similar comments can be made also for the trends of both G’ and G’’, see Figure 3b,c; all of the results suggest the good dispersion of particles and unchanged composite processability in comparison to that of neat PLA.

**Figure 4:**
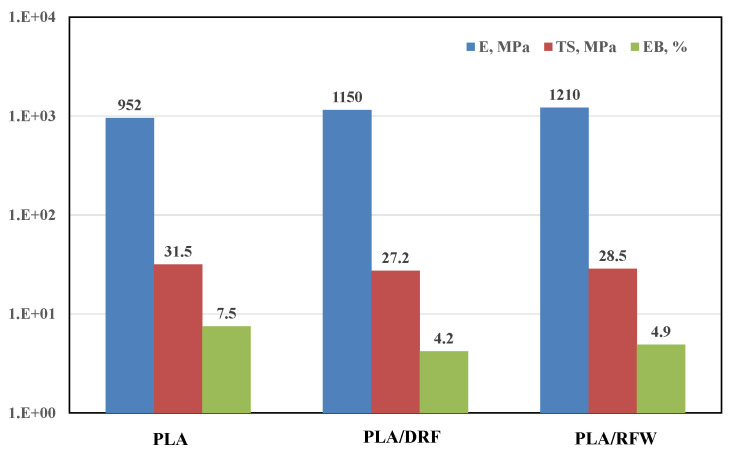
Main mechanical properties, i.e., elastic modulus, E; tensile strength, TS; and elongation at break, EB, of neat PLA and PLA containing 10%wt. of DRF and RFW.

In Figure 4, the main mechanical properties, i.e., elastic modulus, tensile strength, and elongation at break of neat PLA and PLA containing DRF and RFW, are reported. It can be noticed that the addition of both DRF and RFW leads to a slight increase in the values of elastic modulus, while the tensile strength and elongational at break values remain almost unchanged. This result again suggests good particle dispersion and the formulation of biopolymer-based composites with suitable mechanical behavior for packaging applications.

**Figure 5:**
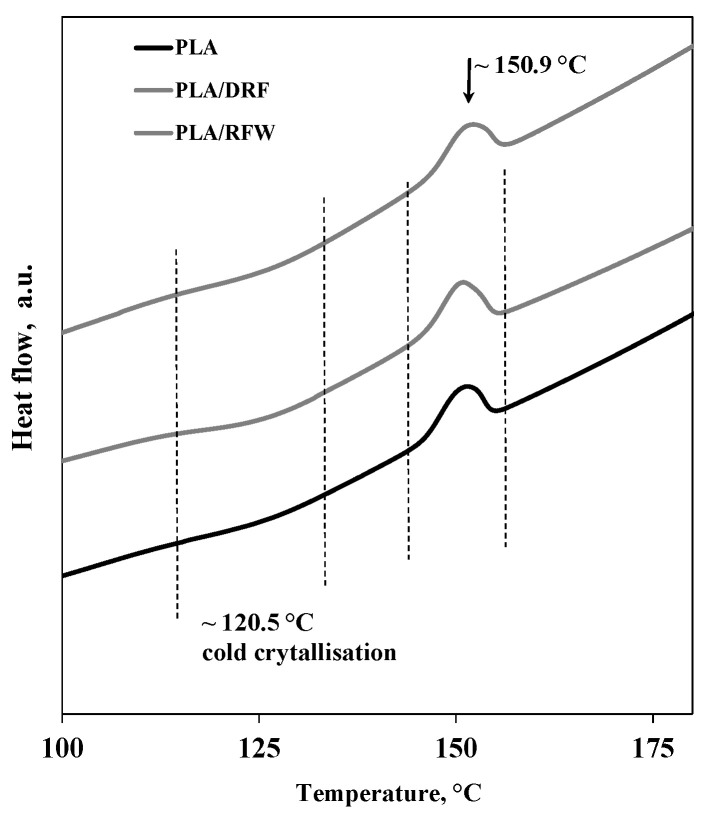
DSC trends of neat PLA and PLA containing 10%wt. of DRF and RFW.

To investigate the thermal behavior of neat PLA and PLA-based composites, differential scanning calorimetry was carried out, which obtained DSC traces, and a summary of important DSC data is plotted in Figure 5 and Table 2, respectively. The DSC trends and data clearly highlight that the presence of both DRF and RFW does not significantly modify the thermal behavior of PLA. The values of melting temperatures and fusion enthalpies remain almost unchanged by adding DRF and RFW, see Table 2.

**Figure 6:**
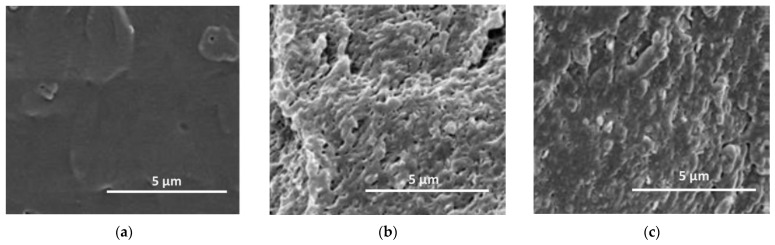
SEM images of (**a**) neat PLA, (**b**) PLA/DFR, and (**c**) PLA/RFW.

The morphology of neat PLA and PLA containing DFR and RFW has been investigated by SEM analysis, and in Figure 6a–c, representative micrographs are reported. It is evident that neat PLA morphology appears more smooth (see Figure 6a), and as expected, the morphology of the composites appears less regular due to the presence of both DRF and RFW (see Figure 6b-c). The observed morphology of composites is typical for all PLA-based materials containing lignin-cellulose derivatives. 

### 2.3. Photo-Oxidation Resistance of PLA/DFR and PLA/RFW

The photo-oxidation behavior of neat PLA and PLA containing both DRF and RFW has been evaluated through the accelerated UV-light exposure of thin films using UVB-lamps, having broad emission in 280–330 nm and an emission peak maximum at 313 nm.

**Figure 7:**
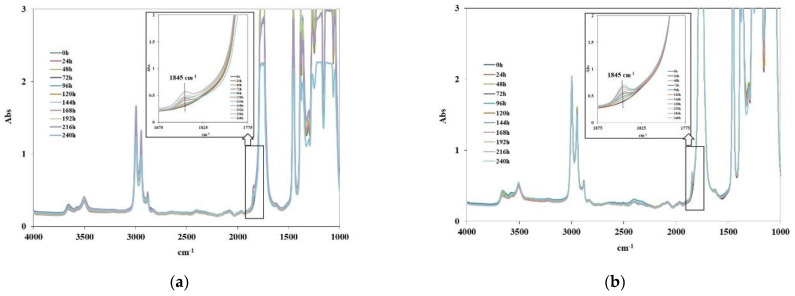

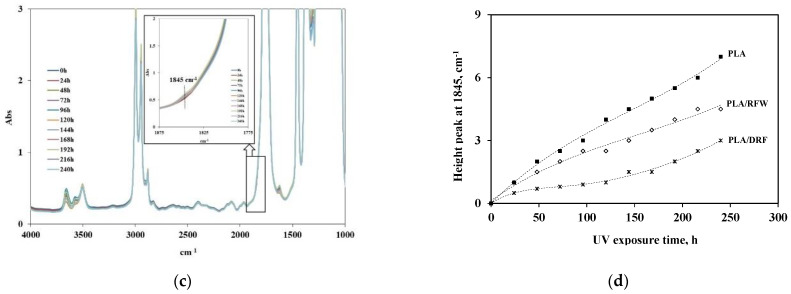
FTIR spectra of (**a**) PLA, (**b**) PLA/DRF, and (**c**) PLA/RFW at different photo-oxidation times. (**d**) Height peak at 1845 cm^−1^ for neat PLA and investigated composites as a function of the photo-oxidation times.

According to the literature, the predominant degradation pathway in the presence of humidity of PLA is hydrolysis, while upon UVB exposure, PLA chains undergo a random chain scission, leading to the formation of anhydride groups having an IR-absorption peak around 1845 cm^−1^ [21,22]. Therefore, monitoring the height of peak abound 1845 cm^−1^ can be profitable following the photo-oxidation behavior of neat PLA and PLA-based composites. The obtained results are shown in Figure 7, and it is clear that the presence of both DRF and RFW have a beneficial effect on the photo-oxidation behavior. The anhydride accumulation is slower for PLA/RFW, and even more for PLA/DRF, in comparison to the anhydride accumulation of neat PLA. It seems that DRF works better in the protection of PLA against UVB exposure, and this could be understood considering that the DRF are not subjected to oil extraction, and, probably, large amounts of oil and antioxidant moieties are still present.

## 3. Discussion

This work aims to valorize two different kinds of *Bulgarian Rosa Damascena Mill.* as suitable fillers for PLA to formulate materials for packaging applications. Dry rise flower (DRF) and rose flower waste (RFW), before and after essential oil extraction, respectively, were introduced successfully to PLA by melt mixing. Both DRF and RFW were subjected, first, to natural drying to eliminate contained water molecules and second to milling to obtain a homogeneous power. As documented elsewhere, the rose flower contains numerous constituents, more than 40 different constituents, and rose flower waste, after essential oil extraction, has been proposed as a food additive in animal husbandry or phytonutrients. Here, rose waste was used as a suitable additive for the formulation of PLA-based packaging films.

The obtained results by spectroscopy and elemental analyses suggest that the DRF and RFW are very similar in terms of lignin–cellulose contents, and the industrial oil extraction process removes oils constituents, which are not crucial for the formulation of PLA-based composites. Interestingly, the DRF and RFW show similar scavenging ability and efficiency, and this result again suggests the possibility to consider the RFW as valuable fillers.

Therefore, PLA/DFR and PLA/RFW show very similar thermal, rheological, and mechanical properties, because, as is known, these properties are mainly influenced by the dimension of particles, especially considering the very similar nature, in terms of lignin–cellulose composition, of both DFR and RFW particles. However, both DRF and RFW slightly increase the PLA rigidity, and this does not have a negative effect on system processability. As expected, the presence of DRF and RFW slightly decreases the fusion enthalpy, suggesting a slight decrease in PLA crystallinity. The micrometric particles are not able to explain the nucleating effect on PLA; rather, they decrease the overall crystallinity.

Interestingly, both DRF and RFW can slow down the PLA upon UVB light, and this is an important result considering that, usually, the micro/nanoparticles, added to the PLA, catalyze its degradation phenomenon. However, although both DRF and RFW contain reduced amounts of essential oils and antioxidant molecules, they have a beneficial effect on the photo-oxidation resistance of PLA. This result is very important considering the potential application of PLA/DRF and PLA/RFW as valuable materials for packaging applications.

All of the results highlight the fact that both DRF and RFW have very similar properties and exert similar effects on the properties of PLA-based composites. This makes both DRF and RFW good candidates for the formulation of new-generation packaging films, especially considering their circular principles, which are requested by current legislation and public opinion.

## 4. Materials and Methods

### 4.1. Materials

The polylactic acid (PLA) used in this work is a commercial extrusion sheet grade supplied by NatureWorks (Nebraska, USA, named PLA 2002D) with average number molecular weight of about 121,000 g/mol, a ratio 96% L-lactide to 4% D-lactide units, and a melt flow index 6 g/10 min (230 °C, 2.16 Kg). Prior to processing, PLA pellets were dried at 70 °C under a vacuum overnight.

Two different kinds of rose flower particles, coming from *Bulgarian Rosa Damascena Mill.,* specifically, dry rose flower (DRF) and rose flower waste (RFW), were used in this work to prepare composites based on PLA. Dry rose flower was obtained by subjecting fresh roses to natural drying for 1 month (no direct sunlight) to eliminate gradually contained water molecules and was then subjected to manual milling, obtaining particles with dimensions ca. 450 ± 51 μm. Rose flower waste (RFW) is the residual flower part after essential oil extraction at industrial level. Obtained RFW was subjected to natural drying for 1 month (no direct sunlight) to eliminate gradually contained water molecules and then was subjected to manual milling, obtaining particles with dimensions ca. 475 ± 53 μm.

### 4.2. Processing

Neat PLA, PLA/DRF, and PLA/RFW samples were processed in a Brabender mixer at 170 °C for 5 min at 50 rpm. The samples were obtained as a film, in a Carver press at 170 °C and pressure 1550 psi (ca. 10.1 MPa), for 2 min.

### 4.3. Characterizations:

- *Accelerated photo-oxidation:* Photoxidation was carried out using a Q-UV/basic weatherometer (from Q-LAB, USA) equipped with UVB lamps (313 nm). The weathering conditions consisted of a continuous light irradiation at T = 70 °C.
- *FTIR spectroscopy:* A Fourier transform infrared spectrometer (Spectrum One, Perkin Elmer) was used to record IR spectra using 16 scans at a resolution of 1 cm^−1^. The progress of the photo-oxidation degradation of the samples was followed by FTIR analysis monitoring of the variations of carbonyl range (1800–1600 cm^−1^) in time, using Spectrum One software.
- *Measurement of particle size:* The size of the three different biochar particles was measured using a Malvern Mastersizer 2000 granulometer with an ultrasound treatment. The Mastersizer 2000 granulometer was equipped with a Malvern Hydro 2000 MU, which uses a stirrer for the dispersion of 1 gr of samples into 800 mL of deionized water. All of the analysis was carried out at a stirrer velocity of 2000 rpm, after 5 min of sonication.
- *Measurement of particle composition:* The elemental analysis, i.e., the determination of carbon, hydrogen, and nitrogen, has been performed by means of a TruSpec CHN LECO CHN 628 (ASTM D5373) analyzer.
- *Tensile test:* Tensile tests were carried out using a universal testing machine (Instron model 3365, UK), according to the ASTM D882 method, on rectangular films.
- The tests were performed using a tensile speed of 1 mm/min for 1 min to evaluate the Young’s modulus, and then the velocity was increased to 10 mm/min until sample breakage.
- *Rheological analysis:* Rheological tests were performed using a stress-controlled rheometer (ARES G-2) in parallel plate geometry (plate diameter 25 mm). The complex viscosity (η*), storage (G’) and loss (G’’) moduli were measured under frequency scans from ω = 10^−1^ to 100 rad/s at T = 150 °C. The strain amplitude was γ = 5%, whose preliminary strain sweep experiments proved to be low enough to be in the linear viscoelastic regime.
- *Differential scanning calorimetry:* The calorimetric data were evaluated by differential scanning calorimetry (DSC), using a Perkin–Elmer DSC7 calorimeter, at a scanning rate of 5 °C/min.
- *Scanning electron microscopy:* The SEM analysis was performed on cryogenically fractured and gold sputtered surfaces of thin-compression-molded samples using a Philips (Netherlands) ESEM XL30 scanning electron microscope.
- *Radical scavenging efficiency:* The 1,1-diphenyl-2-pycryl (DPPH, supplied by Sigma Aldric) free radical scavenging assay was carried out [23,24]. First, a methanol solution of DPPH (10^−4^ M) was prepared; then, 1 mg of solid was placed in 2 mL of this solution for 24 h at 25 °C. The solutions were kept at 25 °C during the time required by the measurement. Then, the supernatant liquid was removed, and the UV-vis spectrum was recorded at different step times up to 24 h in a Beckmann DU-800 spectrometer. Spectra were recorded on a spectrophotometer equipped with a Peltier temperature controller. Moreover, the DPPH assay was also performed in the presence of different amounts of BC, adding 1, 2, 5, and 10 mg of solid in the methanol solution of DPPH (10^−4^ M), recording spectra after 24 h. Scavenging activities were determined from the drop in absorbance at 517 nm of each sample compared with that of the DPPH solution in the absence of contact with the material. Scavenging efficiency values were calculated by Equation (1):
  (1) $$\mathrm{Radical}\;\mathrm{Scavenging}\;\mathrm{Efficiency}\;(\%)=\frac{A-B}{A-0.1}\times 100$$
  where *A* is the absorbance at 517 nm of the DPPH solution and *B* of the DPPH solutions after contact with the solid.

## 5. Conclusions

In this work, considering the circular principles of materials, PLA containing DRF and RFW has been used as a suitable filler for the formulation of packaging films. Although the RFW has been subjected to essential oil extraction, these particles show similar properties to that of DRF.

PLA/DRF and PLA/RFW systems show very similar rheological, mechanical, and thermal behavior, and the presence of both DRF and RFW does not negatively influence the overall properties of PLA films.

Therefore, both DRF and RFW have a beneficial effect on the photo-oxidation resistance of PLA, exerting, albeit reduced, protection ability against UVB exposure. This result is very important because usually different particles added to PLA have a catalytic effect on its degradation phenomena. However, both DFR and RFW could be considered good candidates for the formulation of biopolymer-based films for packaging applications, especially considering their beneficial effect on PLA pho-oxidative degradation.

## Figures and Tables

**Table 1:** Elemental composition and particle dimension, after milling, of dry rose flower (DRF) and rose flower waste (RFW).

	%C	%H	%N	d, μm
DRF	52.54 ± 0.8	3.63 ± 0.04	7.34 ± 0.04	450 ± 51
RFW	63.10 ± 0.2	2.69 ± 0.02	6.54 ± 0.06	475 ± 53

**Table 2:** Melt temperature, T °C, and fusion enthalpy, ∆H, on the second heating scan for neat PLA and PLA containing DRF and RFW.

	Melting Temperature, °C	∆H = ∆Hf − ∆Hcold cryst, mW
DRF	150.5	6.4
PLA/DRF	151.8	3.7
PLA/RFW	151.5	4.1

## Data Availability

Not applicable.

## Sample Availability

Samples of the compounds are available from the authors.
